# Investigation of optical and electrical properties of novel 4T all perovskite tandem solar cell

**DOI:** 10.1038/s41598-022-10513-4

**Published:** 2022-04-25

**Authors:** Mahsa Moradbeigi, Mohammad Razaghi

**Affiliations:** 1 Department of Physics, Faculty of Science, University of Kurdistan, Sanandaj, Iran; 2 Department of Electronics and Communication Engineering, Faculty of Engineering, University of Kurdistan, Sanandaj, Iran

**Keywords:** Photonic devices, Solar cells

## Abstract

In this paper, a combined three-dimensional (3D) optical-electrical simulation of non-pb and flexible four-terminal (4T) all perovskite tandem solar cell (APTSC) is presented. In this structure, polyethylene terephthalate (PET) is used as substrates, while the top sub cell has a $MAGeI_{3}$ absorber layer and the bottom sub cell has a $MASnI_{3}$ absorber layer. This structure is used as a reference in this paper and the optical and electrical properties of it are investigated using the finite element method (FEM). It is shown that this structure has a total power conversion efficiency (PCE) of $24.65\%$. Then, the elimination of the buffer layer and the addition of antireflection layer (ARL) strategies, as well as the use of periodic nano-texture patterns, are used to increase the reference structure’s total PCE. A free-buffer layer tandem device is presented to minimize the parasitic absorption. While the total PCE is improved by $1.14\%$ in this case, one of the fabrication steps is also eliminated. A plasma-polymer-fluorocarbon (PPFC) coating layer is suggested as ARL on the substrates of both sub cells to reduce reflection loss. With optimized these layers thickness, total PCE is increased by $12.76\%$. Because the PPFC layer is hydrophobic, the top surface of two sub cells in this structure has self-cleaning characteristic. As a result, this device offers long-term moisture resistance. Finally, the best structure in terms of the maximum total PCE is presented by increasing optical path-length utilizing nano-photonic and nano-plasmonic structures. The final structure is offered as a 4T tandem solar cell (TSC) that is environmentally friendly, extremely flexible, and has self-cleaning capability, with a total PCE of $30.14\%$, which is greater than the total PCE of the reference structure by $22.27\%$.

## Introduction

Recently, there has been an increase in research on organic-inorganic metal-halide (oxide) perovskite solar cells (PSCs) due to their suitable bandgap tunability from 1.18 to 2.3 eV^1^ with large absorption coefficients, easy fabrication steps with low temperature processing, and low cost due to their cheap raw materials^2–4^. Within 12 years, the power conversion efficiency (PCE) of single-junction PSCs has risen from less than $4\%$ to more than $25\%$^5^, approaching the Shockley–Queisser theoretical PCE limit of 31–$33\%$^6^. Therefore, to overcome this limitation, two-junction (tandem) architecture is introduced^7^.

Tandem solar cells (TSCs) integrate two sub cells whose absorber material of each sub cell originates from anomalous materials or similar structural (chemical) formula materials with different band gaps^7,8^. The tandem device is manufactured by adding a semitransparent front cell with a wide band gap (WBG) absorber material on top of a rear cell with a narrow band gap (NBG) active layer. The WBG solar cell, referred to as the top sub cell, harvests short-wavelength photons, while the NBG solar cell, referred to as the bottom sub cell, absorbs long-wavelength photons. As a result, TSCs achieve higher PCE by utilizing a wider range of sunlight. When combined with Si^9,10^, CIGS^6,11^, CZTS^12^, NBG perovskite^13,14^, colloidal quantum dot^15,16^, organic^17^ bottom sub cells, WBG perovskite absorber materials are excellent choices for the top sub cells. Because of the superior optical and electrical properties of perovskite materials, WBG/NBG perovskite TSCs or all perovskite TSCs (APTSCs) can be offered for dominating the PCE limit of single junction PSCs. Thin-film APTSCs are available in two configurations: monolithically integrated two-terminal (2T) architecture and mechanically stacked four-terminal (4T) setup. The optical and electrical coupling between the top and bottom sub cells is established in the 2T structure, whereas the optical coupling between two sub cells is established in the 4T structure, and these sub cells are electrically independent. Since the electrical coupling of two sub cells is series in the 2T structure, the current of each sub cell needs to be identical. That is defined as current matching conditions^18^. Hence, material choices are limited to satisfy this condition in the 2T architecture. Mechanically stacked 4T device, in contrast, is not limited by current matching issue. The first 4T APTSC with a PCE of $19.1\%$ had been reported^8^. Later on, Tong et al. achieved an impressive PCE of $25.4\%$ for a 4T tandem architecture using a 1.63 eV bandgap $Cs_{0.05}FA_{0.8}MA_{0.15}PbI_{2.55}Br_{0.45}$ perovskite as the absorber material of top sub cell and a 1.25 eV bandgap $(FASnI_{3})_{0.6}(MAPbI_{3})_{0.4}$ perovskite as the active layer of bottom sub cell^19^. Recently, Nejand et al. reported a 4T APTSC based on the $Cs_{0.1}(MA_{0.17}FA_{0.83})_{0.9}Pb(I_{0.83}Br_{0.17})_{3}$ perovskite as the WBG absorber and $FA_{0.8}MA_{0.2}Sn_{0.5}Pb_{0.5}I_{3}$ perovskite as the NBG absorber. The resulting device had a PCE of $23\%$^13^.

Despite the high PCE of lead-containing PSCs, the toxic nature of Pb compounds is a major concern that prevents their widespread use. As a result, a lead-free perovskite material alternative is proposed as a suitable solution. Despite previous research confirming that Ge and Sn could be a potential candidate for replacing Pb in Pb-based halide perovskites due to their non-toxic nature, earth abundant element, and environmentally friendly (eco-friendly) characteristics^20–26^, single junction Ge-based and Sn-based halide perovskite solar cells suffer from low PCE when compared to Pb-based halide perovskite solar cells^27,28^. Using Sn-based and Ge-based PSCs in tandem structure can therefore provide higher PCE than single junction Pb-based PSCs.

The goal of our current work is to present a TSC device that is low-cost, high-efficiency, and has excellent mechanical flexibility, as well as self-cleaning and eco-friendly characteristics. A numerical modeling of 4T Ge-based perovskite/Sn-based perovskite TSC is performed in this work using a three-dimensional (3D) finite element method (FEM). We introduced eight different cases of free-Pb 4T APTSC with polymer material substrates for each sub cell with the top sub cell including $MAGeI_{3}$ perovskite as the WBG absorber layer and the bottom sub cell including $MASnI_{3}$ perovskite as the NBG active layer. First, the optical and electrical properties of the $MAGeI_{3}$ PSC and $MASnI_{3}$ PSC in the single junction structure are investigated. Then, a 4T $MAGeI_{3}$/$MASnI_{3}$ perovskite TSC as reference structure (case (I)) is designed and analyzed. Following that, we proposed a semi-transparent perovskite top sub cell without a metal oxide buffer layer as a case (II) to reduce parasitic absorption and eliminate one of the fabrication procedures. In cases (III) and (IV), plasma-polymer-fluorocarbon (PPFC) is employed as an antireflection layer (ARL) to reduce the reflection loss of incident light from the surface of two sub cells. It is optimized in thickness for each sub cell, and the optical properties of these cases are investigated. In cases (V)-(VIII), we examined light-trapping structures based on nano-texture structures to improve absorption ability in both absorber layers. The absorption properties of WBG and NBG perovskite layers, as well as total reflection loss, are investigated and compared for all presented cases. To conclude, the electrical properties of each sub cell and tandem device are analyzed for all of the considered cases, and the optimal structure is introduced to get the highest possible total PCE.

## Results

**Figure 1:**
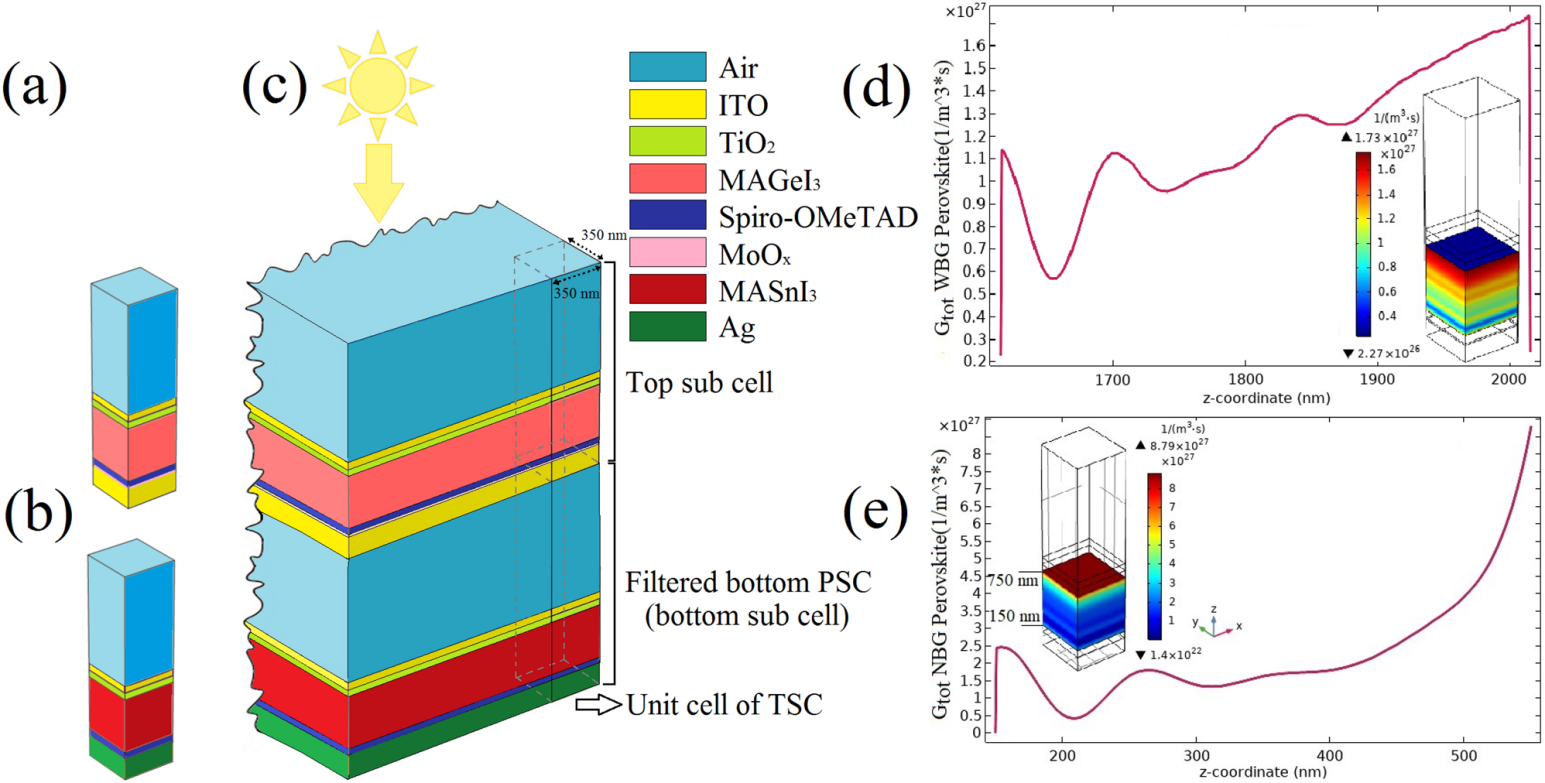
A schematic of the unit cell of single junction WBG PSC (**a**), stand-alone NBG PSC (**b**) and 4T APTSC (**c**). The total charge carrier generation rate versus perovskite layer length (along with the z-coordinate) and 3D map of total generation rate for single junction WBG PSC (**d**) and stand-alone NBG PSC (**e**).

To design eco-friendly 4T APTSC, we considered $MAGeI_{3}$ (with $E_{g} = 1.9$ eV)-based PSC as the top sub cell and $MASnI_{3}$ (with $E_{g} = 1.3$ eV)-based PSC as the bottom sub cell. The configuration of semi-transparent top sub cell consists of the following layers from top to bottom: the indium tin oxide (*ITO*, 50 nm) as the transparent conductive oxide (*TCO*), titanium dioxide ($TiO_{2}$, 50 nm) as the electron transport material (*ETM*), $MAGeI_{3}$ (400 nm) as the WBG perovskite absorber material, *Spiro*–*OMeTAD* (50 nm) as the hole transport material (*HTM*), molybdenum oxide ($MoO_{x}, 10$ nm) as the buffer layer and the *ITO* (105 nm) as *TCO*. The bottom sub cell layers from top to bottom are the *ITO* (50 nm) as the *TCO*, $TiO_{2}$ (50 nm) as the *ETM*, $MASnI_{3}$ (400 nm) as the NBG perovskite active layer, *Spiro*–*OMeTAD* (50 nm) as the *HTM* and the *Ag* (100 nm) as the back-contact. In order to design a flexible solar cell, we considered polyethylene terephthalate (PET) as the substrate of two sub cells. One of the proposed methods for fabrication of each sub cell is as follows: a thin film of $TiO_{2}$ layer is deposited on the PET/TCO substrates by atomic layer deposition^29^. After the spin coating of the WBG or NBG perovskite thin film^25^, a thin film of *Spiro*–*OMeTAD* is spin-coated on absorber layer of each sub cell^30^. Next, a 100 nm *Ag* back-contact is thermally evaporated on *HTM* of bottom sub cell^31^. Before the ITO sputtering as rear electrode of top sub cell, a 10 nm $MoO_{x}$ buffer layer is thermally evaporated on *HTM* of semi-transparent top sub cell to protect the *HTM* layer against sputtering damage^32^. After that, the sub cells were integrated in the mechanically stacked 4T tandem architecture. The schematic of the proposed single junction WBG PSC, stand-alone NBG PSC and 4T tandem structure is shown in Fig. 1a–c. Figure 1d,e show the profiles of total generation rate ($G_{tot}$) versus perovskite layer length, as well as 3D maps of $G_{tot}$ in the entire absorber layer for both WBG and NBG PSCs that were not placed in tandem configuration. The simulation results show that the total charge carrier generation rate reaches a maximum ($G_{totmax}$) in the perovskite/ETM junction of each PSC. The $G_{totmax}$ is achieved $1.73 \times 10^{27}\;{{\text {m}}}^{3}\;{\text {s}}$ and $8.79 \times 10^{27}\;{{\text {m}}}^{3}\;{\text {s}}$ at the WBG and NBG perovskite layers, respectively. These results have been validated by replacing the $MAPbI_{3}$ active layer with $MASnI_{3}$ and $MAGeI_{3}$ materials in the previous study^33^. Also, the glass/TCO is changed with PET/TCO substrate. For this purpose, *n* and *k* of these materials in previous simulation^33^ replaced with new parameters^25,34–36^.

**Figure 2:**
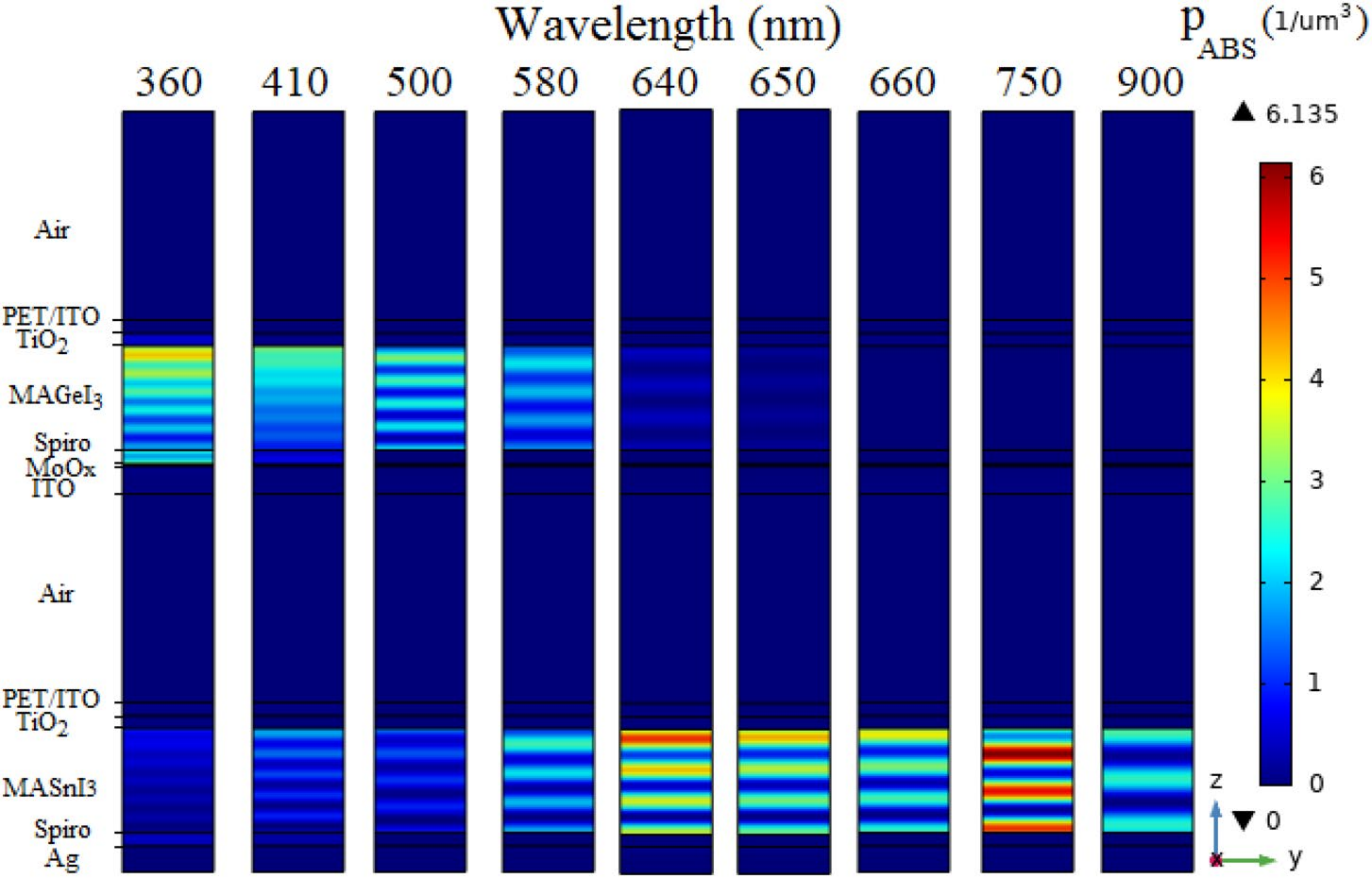
The absorption density profiles of the proposed tandem architecture shown in Fig. 1c for several wavelengths.

The absorption density ($p_{ABS}$) profiles of the proposed 4T tandem structure (Fig. 1c) is presented in Fig. 2 for $\lambda$ of 360, 410, 500, 580, 640, 650, 660, 750 and 900 nm. Because the $E_{g}$ of these absorber layers is smaller than that of other layers, the majority of the incident solar spectrum is absorbed in the perovskite active layer of each sub cell. Because of the WBG perovskite material bandgap energy, it can absorb photons with energies of $>1.9$ eV, which corresponds to photons with wavelengths of $<660$ nm. Sunlight is filtered by passing it through the semi-transparent top sub cell and absorbing a portion of it. After that, the filtered solar light is guided to the bottom sub cell. As a result, the bottom PSC in the tandem structure is referred to as the “filtered bottom PSC”. The NBG perovskite layer ($E_{g}=1.3$ eV) can absorb a wider range of sunlight wavelengths. $MASnI_{3}$ perovskite material absorbs approximately the photons with wavelengths of $<1000$ nm. Maximum absorption of the tandem structure occurs in the active layer of the bottom sub cell for wavelengths exceeding 660 nm. Given that only light absorption in the absorber layer of both sub cells generates electron–hole pairs, absorbed light in the alternative layers is defined as parasitic absorption.

**Figure 3:**
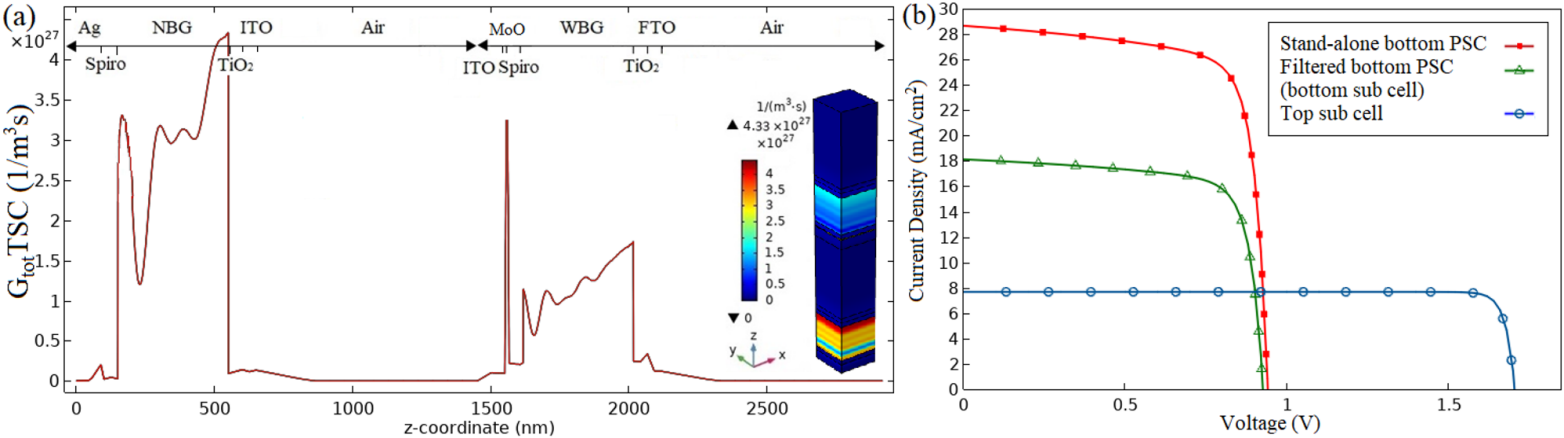
The total charge carrier generation rate profile (**a**) and the current density-voltage characteristics of the top sub cell, filtered and stand-alone bottom PSCs (**b**) for proposed structure presented in Fig. 1c.

Figure 3a illustrates the 1D format of the $G_{tot}$ profile versus device length as well as the 3D format of this parameter in the overall structure of the proposed 4T tandem architecture. The constituent layers of the proposed structure are also shown in the z-coordinate. The maximum value of the total photogeneration rate of WBG and NBG perovskite layers in the tandem structure is obtained in top of each absorber layer of two sub cells, as in single junction structures. The $G_{totmax}$ have reached $1.73 \times 10^{27}{/{\text {m}}^{3}\;{\text {s}}}$ and $4.33 \times 10^{27} /{\text {m}}^{3}\;{\text {s}}$ at the WBG and NBG absorber layers, respectively. The value of $G_{totmax}$ for filtered NBG perovskite layer is greater than the $G_{totmax}$ value for WBG perovskite layer because of the wider range of absorption in $MASnI_{3}$ material compared to $MAGeI_{3}$ material as shown in Fig. 2. Peaks in the buffer and other layers depicted in the Fig. 3a are not involved in the photogeneration rate and are only considered as parasitic absorption. As expected, the $G_{tot}$ value of the WBG perovskite layer in tandem structure is the same as that value in the single junction structure shown in Fig. 1d. The $G_{tot}$ value of the filtered NBG perovskite layer is lower than that of the stand-alone NBG perovskite layer due to partial sunlight absorption by the semi-transparent top sub cell (comparison of Figs. 1e and 3a). The current density-voltage (J–V) curve of the top, the filtered and the stand-alone bottom PSC are depicted in Fig. 3b. Since the electrical characteristics of the top sub cell in the tandem architecture are similar to the top PSC in the single junction structure, the term “top PSC” is used for both the tandem and the single junction structures. The $J_{sc}$, $V_{oc}$, *FF* and *PCE* values have been obtained $7.68\;{\text {mA}}/{\text {cm}}^{2}$, 1.70*V*, 0.92 and $12.00\%$ for semi-transparent top PSC and $28.66\;{\text {mA}}/{\text {cm}}^{2}$, $0.94\;{\text {V}}$, 0.76 and $20.37\%$ for stand-alone bottom PSC, respectively. Due to light absorption in semi-transparent top PSC, the $J_{sc}$, $V_{oc}$, *FF* and *PCE* parameters in the filtered bottom PSC, are decreased to $18.12\;{\text {mA}}/{\text {cm}}^{2}$, $0.93\;{\text {V}}$, 0.75 and $12.65\%$ compared to the stand-alone bottom PSC. The PCE of both the semi-transparent top PSC and the filtered bottom PSC should be considered when calculating the PCE of the proposed 4T tandem structure. The PCE of eco-friendly 4T APTSC with flexible substrates for two sub cells is calculated to be $24.65\%$.

**Figure 4:**
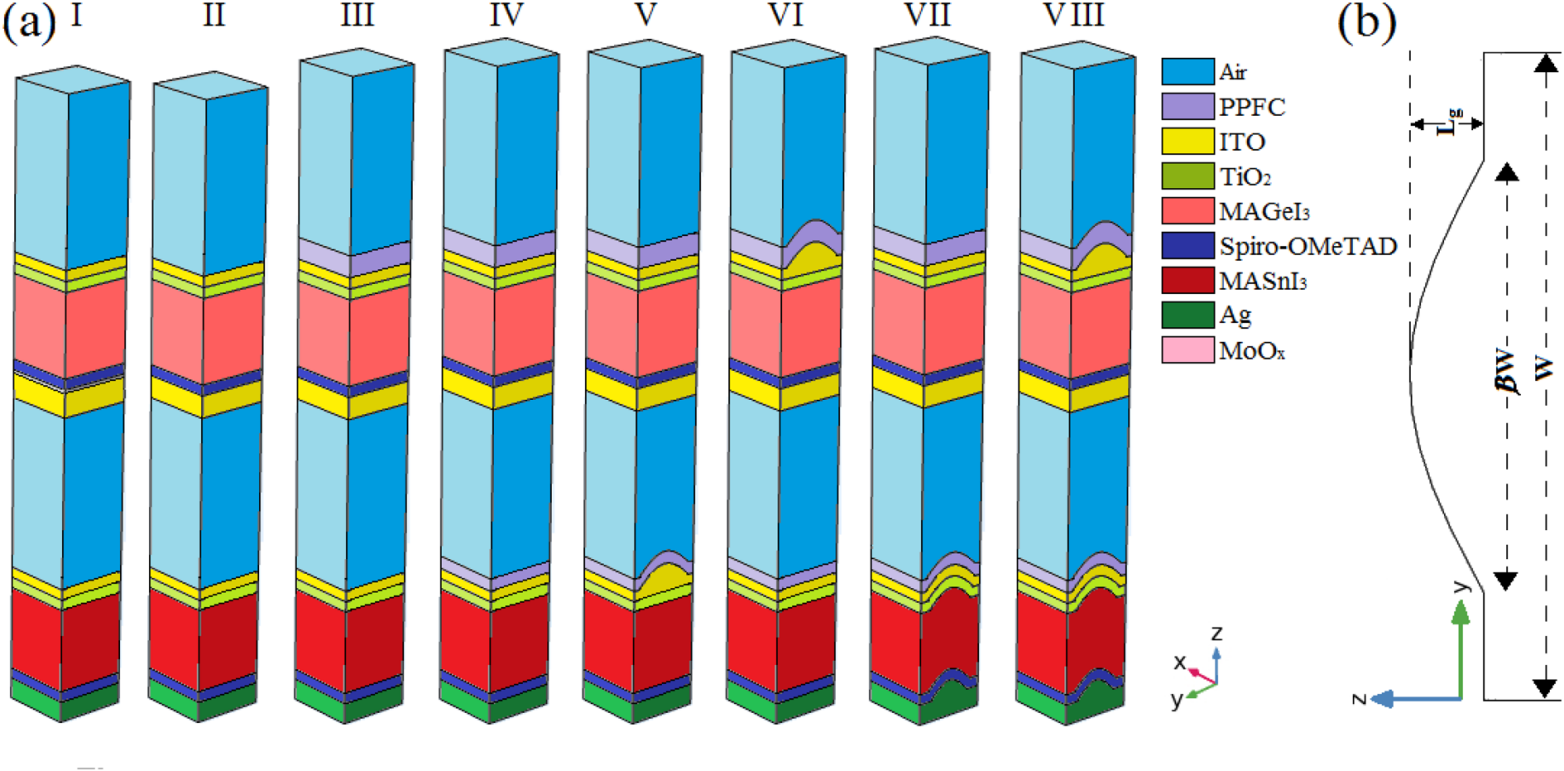
(**a**) 3D schematic of the unit cell of all proposed cases. (**b**) 2D schematic of grating patterns.

To improve the efficiency of this proposed structure, which is introduced as case (I), we presented seven different cases (cases (II)–(VIII)) as shown in Fig. 4a. Case (I) will be used as a reference from now on. The buffer layer is removed in case (II). Cases (III) and (IV) take into account the addition of ARLs. In cases (V)–(VIII), grating patterns are used, and a 2D schematic of this pattern is shown in Fig. 4b. The optical and electrical properties of each case are investigated in the following sections.

### The removal of the buffer layer

A semi-transparent top sub cell is required in the TSC to transmit photons with energies less than the band gap of $MAGeI_3$ to the bottom sub cell. Therefore, a transparent rear-contact must be used in the top sub cell. One of the suggestions is to use a *TCO*. Usually, the sputtering process is applied for the *TCO* deposition. A metal oxide buffer layer between the *HTM* and the *TCO* is placed to prevent ion bombardment damage from sputtering process to the *HTM* layer. Nevertheless, parasitic absorption of this layer reduces the bottom sub cell’s efficiency. Therefore, the PCE of the tandem device falls. Efforts have recently been made to eliminate the buffer layer and direct sputtering of the *TCO* on the *HTM*. The sputter parameters of a 105 nm *ITO* layer, including pressure, sputter power and temperature in the chamber, are mentioned by Bett et al.^30^. By direct sputtering of the *TCO* on the *HTM*, not only parasitic absorption as optical loss will be reduced, but also an extra process step in the fabrication will be omitted. Herein, the optical properties of reference structure (tandem structure with buffer layer) and case (II) (tandem structure with the exclusion of buffer layer) are compared.

**Figure 5:**
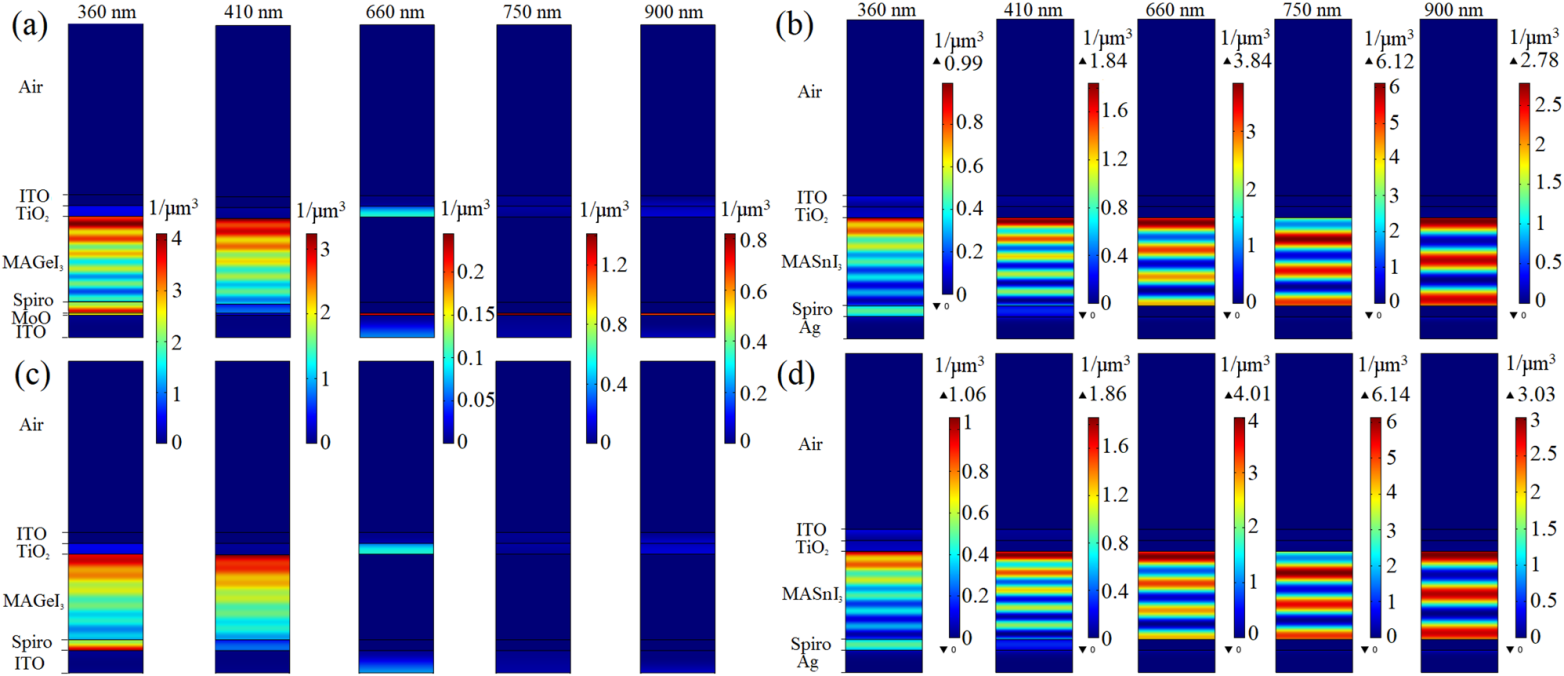
The absorption density distribution in the top sub cell of case (I) (**a**), in the bottom sub cell of case (I) (**b**), in the top sub cell of case (II) (**c**) and in the bottom sub cell of case (II) (**d**) for several wavelengths.

Figure 5 has been demonstrated the absorption density distribution in the top and bottom sub cells of case (I) and case (II) for $\lambda$ of 360, 410, 660, 750 and 900 nm. Figure 5a,c are related to $p_{ABS}$ profiles of the top sub cell with and without the buffer layer, respectively. As shown, the $p_{ABS}$ value of $MAGeI_{3}$ layer is slightly decreased by elimination of buffer layer. Since the refractive index of the buffer material is lower than that of the *Spiro*–*OMeTAD* material, some light is reflected from the HTM/buffer interface. Because the refractive index of *Spiro*–*OMeTAD* material is lower than that of $MAGeI_{3}$ material, it is absorbed in this layer. However, by deleting the buffer layer, the light at the HTM/ITO interface penetrates into the *ITO* layer because the refractive index of *ITO* is greater than the *Spiro*–*OMeTAD* material. Therefore, the $p_{ABS}$ value of the absorber layer of the top sub cell with the presence of the buffer layer is greater than without this layer. Effective absorption occurs in $MAGeI_{3}$ material in these two profiles, which is involved in the production of carriers. The absorption density of other layers is considered as the parasitic absorption density. By comparing Fig. 5a with c, additional parasitic absorption density is observed in the $MoO_{x}$ layer for all wavelengths when the buffer layer is existed. The optical loss is less for $\lambda >660$ nm than for $\lambda <660$ nm. The optical loss is decreased by removing the buffer layer. Although extra parasitic absorption in the top sub cell does not change the absorption of the $MAGeI_{3}$ layer, it can affect the absorption of the $MASnI_{3}$ layer in the filtered PSC. Figure 5b,d refer to the $p_ {ABS}$ maps of filtered PSC with and without the $MoO_{x}$ as the buffer layer, respectively. Herein, efficient absorption happens in the $MASnI_{3}$ layer that leads to the generation of electron–hole pairs. In these figures, the amount of optical loss absorption is also seen to have emerged in other layers. By comparing Fig. 5b,d, we found that the $p_{ABS}$ value in the $MASnI_{3}$ layer is increased with the removal of the buffer layer for $\lambda$ of 360, 410, 660, 750 and 900 nm due to reduced parasitic absorption of semi-transparent top sub cell as shown in Fig. 5a.

**Figure 6:**
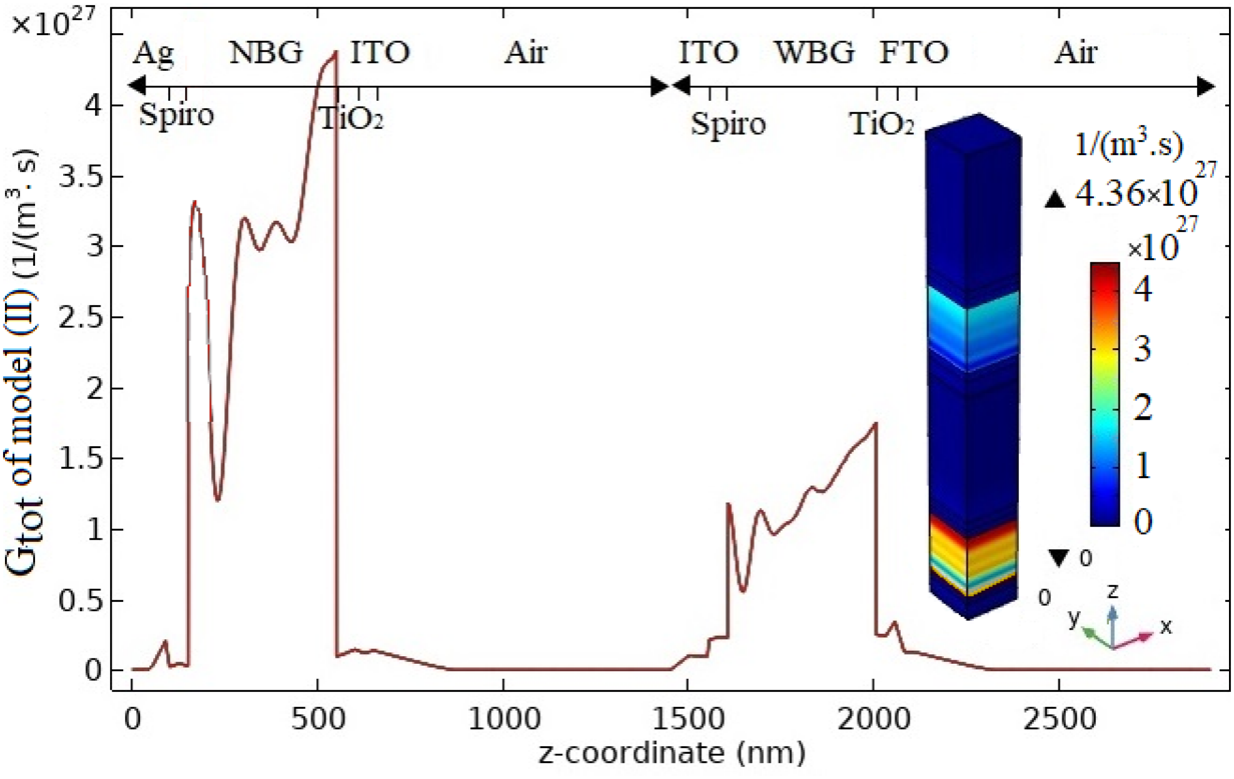
The generation rate profile of charge carrier related to case (II).

The 1D $G_{tot}$ parameter distribution in device length is presented in Fig. 6. This figure also depicts a 3D illustration of this parameter profile in the entire structure. In this curve, the layers of the proposed case (II) are denoted. With the elimination of the metal oxide buffer layer, here, the high peak associated with the buffer layer in Fig. 3a has been omitted due to the deletion of excess parasitic absorption of the buffer layer in the semi-transparent top sub cell. Therefore, the $G_{totmax}$ value of NBG perovskite layer in case (II) is reached to $4.36 \times 10^{27}/{\text {m}}^{3}\;{\text {s}}$ which is increased $0.69 \%$ relative to the $G_{totmax}$ value of NBG perovskite layer in case (I). This parameter for WBG perovskite layer is decreased to $1.71 \times 10^{27}/{\text {m}}^{3}\;{\text {s}}$ which is $1.16 \%$ less than this value in reference case. This is because the reflected sunlight from the HTM/buffer layer contact to the WBG perovskite layer has been eliminated (comparison of Figs. 3a and 6).

### Addition of antireflection layer

Another optical loss in PSC is the Fresnel reflection of sunlight from the top surface of the solar cell. Reflection loss decreases the current density and the PCE. One solution is to use an ARL to reduce the amount of wasted sunlight. In this paper, we utilized a PPFC coating on the plastic substrate as an ARL due to its low refractive index, excellent flexibility, retaining form after repeated bending tests for 10,000 times, nature of superhydrophobic surface, high transmittance over a wide wavelength range, self-cleaning effect, and long-term environmental stability under high humidity, chemicals, and heat^37–39^. A PPFC ARL can be sputtered with mid range frequency wave on PET substrate. In sputtering process, conductive carbon nanotube (CNT)/polytetrafluoroethylene (PTFE) as composite targets is used^38–40^.

**Figure 7:**
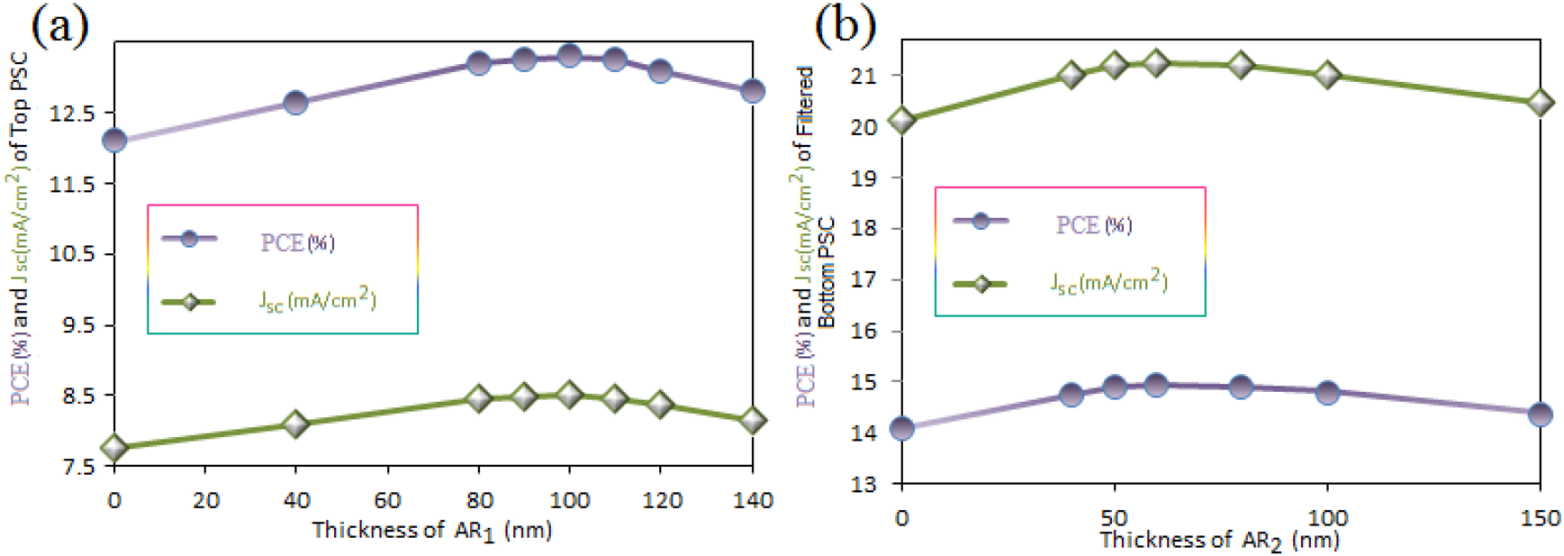
(**a**) Impact of $ARL_{1}$ thickness on $J_{sc}$ and *PCE* parameters of the top sub cell. (**b**) The effect of $ARL_{2}$ thickness on $J_{sc}$ and *PCE* parameters of the bottom sub cell.

Here, impact of adding ARLs on top of each sub cell has been investigated. Thus, the proposed cases (III) and (IV) are compared with case (II) (Fig. 4a). In case (III), a PPFC thin film as $ARL_{1}$ is placed to the PET substrate of just the top sub cell. In case (IV), a PPFC ARL is placed on the top of both sub cells. A PPFC coating on PET substrate of bottom sub cell is introduced as $ARL_{2}$. Figure 7a displays the effect of PPFC $ARL_{1}$ thickness on $J_{sc}$ and *PCE* parameters of the top sub cell. For thickness less than 100 nm, $J_{sc}$ and *PCE* parameters are enhanced by increasing the thickness of $ARL_{1}$. For thickness greater than 100 nm, the value of these parameters is decreased. For 100 nm of PPFC $ARL_{1}$ on PET/ITO substrate of the top sub cell, the maximum $J_{sc}= 8.44\;{\text {mA}}/{\text {cm}}^{2}$ and $PCE= 13.22 \%$ of the top sub cell are observed. In addition, the $J_{sc}$ and *PCE* parameters of the bottom sub cell are increased to $20.03\;{\text {mA}}/{\text {cm}}^{2}$ and $14.00 \%$, respectively, as compared to case (II) (where the $ARL_{1}$ is not used). This occurs as a result of increased light penetration into the tandem structure caused by reduced reflection loss of the top surface of the top sub cell. The effect of $ARL_{2}$ thickness on $J_{sc}$ and *PCE* parameters of the bottom sub cell with a constant thickness of 100 nm $ARL_ {1}$ is shown in Fig. 7b. It shows that the maximum values of $J_{sc}$ and *PCE* parameters of the bottom sub cell are achieved for a thickness of 60 nm. For this thickness, $J_{sc}$ and *PCE* values have been reached $21.16\;{\text {mA}}/{\text {cm}}^{2}$ and $14.83 \%$, respectively.

**Figure 8:**
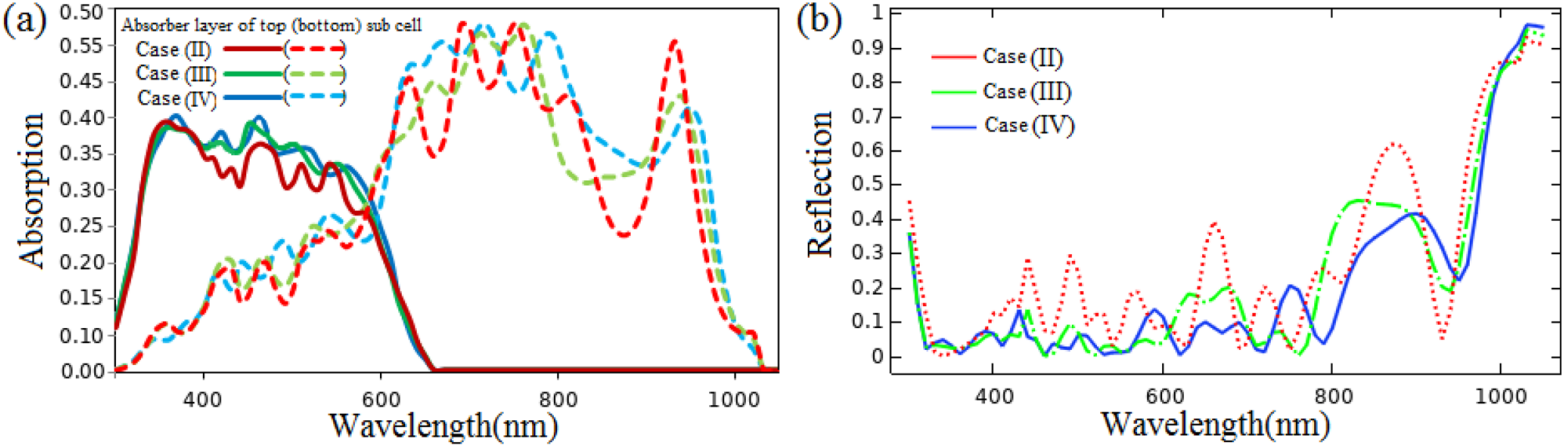
Absorption spectrum of absorber layer of the top and bottom sub cells (**a**) and total reflection spectrum (**b**) for cases (II)–(III).

Figure 8a,b demonstrate the absorption of the active layer of the top and bottom sub cells and the total reflection spectrum in the proposed cases (II), (III), and (IV) for wavelengths between 300 and 1050 nm. As expected, the use of ARLs (blue and green lines in Fig. 8b) has lowered reflection losses at almost all wavelengths when compared to the absence of ARLs (red line in Fig. 8b). The absorption of the $MAGeI_{3}$ active layer is increased at wavelengths spanning from 380 to 620 nm by placing 100 nm PPFC ARL on the PET/ITO substrate of the top sub cell. Moreover, absorption improvement of $MASnI_{3}$ active layer can be observed at wavelengths between 410 and 610, 640–680, 700–790, 840–910, and 950–1010 nm. This is due to that fact that the refractive index of PPFC is closer to that of air than that of PET/ITO. Therefore, the difference between the refractive indices of the top layer of the top sub cell and the air layer is adjusted, which decreases the reflection of the top surface of the top sub cell. As a result, light is penetrated into the tandem architecture (top and bottom sub cells) at these wavelengths, resulting in absorption enhancement in the $MAGeI_{3}$ and $MASnI_{3}$ layers. On the other hand, there is a decrease in absorption of the active layer of the bottom sub cell at wavelengths spanning from 610 to 640, 680–700, 790–840, and 910–950 nm. This is related to an increase in total reflection loss at these wavelengths induced by destructive interference (DI) between reflected light and entering light into the structure. Therefore, a portion of the sunlight in these wavelength ranges did not reach the device. These ranges are affected because they are in the absorption range of the bottom sub cell (comparison of cases (II) and (III)). By comparing case (III) and (IV), as expected, we found that the addition of $ARL_{2}$ does not have a significant effect on the absorption of the $MAGeI_{3}$ active layer. However, in the bottom sub cell, noticeable absorption enhancement occurs at wavelengths between 610 and 730, 780–890, and 950–1000 nm. This is because the total reflection loss from the top of the bottom sub cell is decreased at these ranges by reducing the difference in the refractive index of the top layer of the bottom sub cell with the air layer. The reduction of absorption in several wavelengths is related to the DI of the reflected light with the incoming light to the bottom sub cell which the total reflection loss is increased.

**Figure 9:**
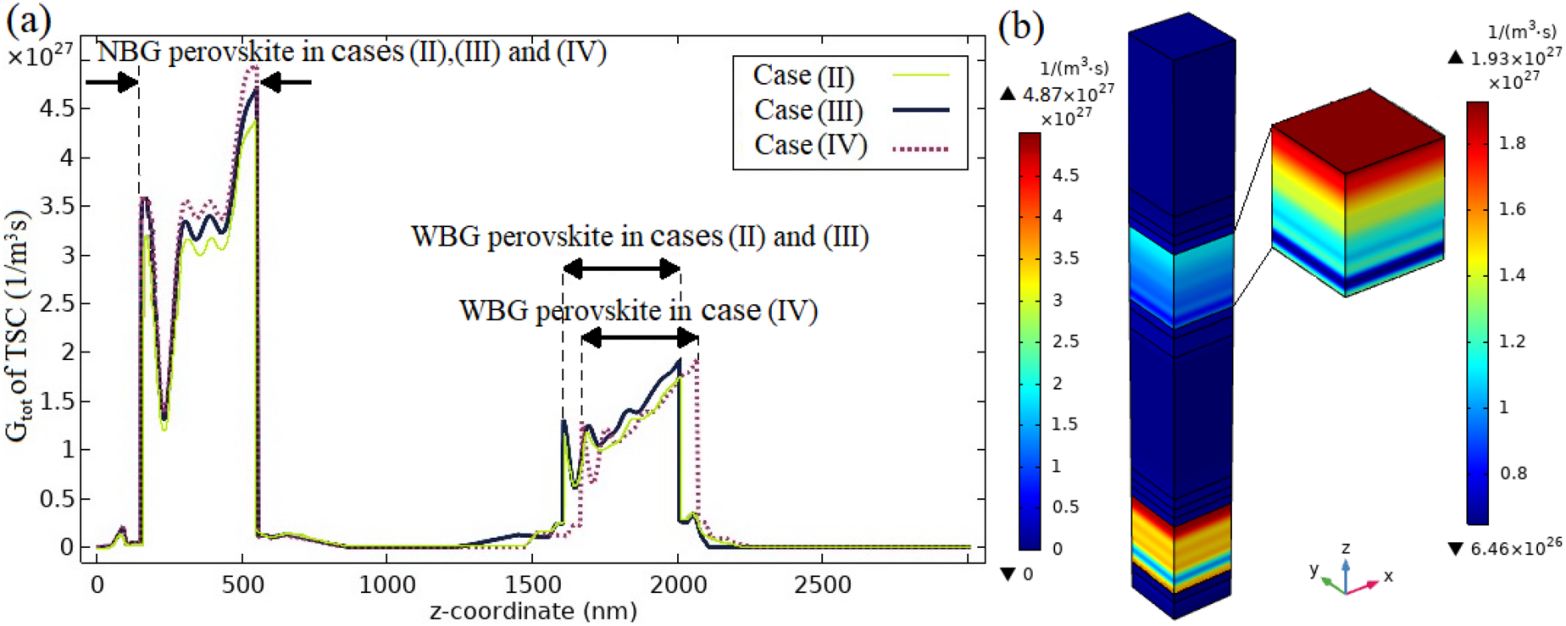
(**a**) 1D format of the photo generation rate distribution regarding cases (II)–(IV). (**b**) 3D profile of photo generation rate for case (IV).

The calculation results of 1D $G_{tot}$ profile along the device length for the proposed cases (II), (III) and (IV) are illustrated in Fig. 9a. The regions corresponding to absorber perovskite layers of each sub cell are marked in this figure for three proposed cases (II)–(IV). By comparing cases (II) and (III), we discovered that the $G_{tot}$ value of the WBG and NBG perovskite layers has been increased by adding 100 nm PPFC ARL ($ARL_{1}$) to case (II). Due to the total reflection loss reduction and effective absorption enhancement in absorber layers of each sub cell which are shown in Fig. 8, generation of electron–hole pairs is enhanced in both sub cells. In case (III), the $G_{totmax}$ value of the WBG and NBG perovskite layers is reached to $1.92 \times 10^{27}/{\text {m}}^{3}\;{\text {s}}$ and $4.69 \times 10^{27}/{\text {m}}^{3}\;{\text {s}}$, respectively. By considering 60 nm of PPFC ARL ($ARL_{2}$) to top of the bottom sub cell of case (III), the $G_{totmax}$ parameter in NBG perovskite is increased to $4.87 \times 10^{27}/{\text {m}}^{3}\;{\text {s}}$. This is related to the reasons that light emission into the absorber layer of the bottom sub cell is enhanced in case (IV) compared to case (III) by lowering surface reflection losses. Therefore, absorption of the bottom sub cell has been boosted and the generation of electron and hole carriers has been increased. The $G_{tot}$ value of the WBG perovskite has remained almost unchanged, with the only difference being the addition of the thickness of $ARL_{2}$ to the z-coordinate (comparison of case (III) with case (IV)). Figure 9b depicts the 3D distribution of $G_{tot}$ parameter for case (IV). The $G_{totmax}$ value of the WBG and NBG absorber layers is improved by $11.56 \%$ and $12.47 \%$, respectively, when compared to case (I), as shown in Fig. 3a. It’s because ARLs were placed on top of both sub cells to minimise reflection loss.

### Periodic nano-texture structures to improve light absorption

Many techniques have been proposed to take advantage of nano-texture structures for PCE enhancement in PSCs^33,41–44^. These structures, known as nano-photonic and nano-plasmonic structures, can be used on dielectric and metal surfaces. Nano-photonic structures can reduce reflection losses, enhance the optical path-length by light scattering and couple the incident light with waveguide-modes into the semiconductor. Nano-plasmonic structures with a size less than 50 nm confine the sunlight in the absorber layer by using mostly near-field effects, and operate as a scatterers with a size larger than 50 nm, which enhance the optical path-length^45^. The scattering mechanisms of nano-texture structures are discussed for PSC in^41,42^. Soft lithography and nanoimprint lithography methods can be applied to fabricate nano-texture structures^46–48^.

**Figure 10:**
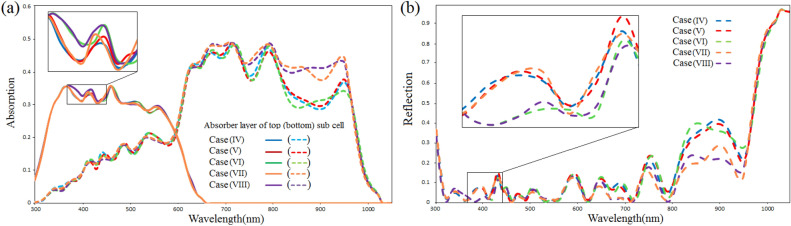
The spectrum of the top and bottom sub cells absorption and total reflection of the proposed cases (IV)–(VIII).

For light absorption enhancement in the case (IV), we have presented cases (V)–(VIII) using the nano-texture structures. The effect of nano-photonic structures on the optical properties of case (IV) has been investigated in cases (V) and (VI) by adding nano-texture structures to the top of each sub cell. In case (V), nano-texture structures have been applied on top of the ARL and TCO layer of the bottom sub cell. The nano-photonic structure above ARL and TCO layer of the top sub cell has been used in case (VI). In cases (VII) and (VIII), both nano-photonic and nano-plasmonic structures have been used to improve the light-capturing ability. In case (VII), nano-texture structures have been designed on top of all layers of the bottom sub cell, including the back-contact/HTM interface. In case (VIII), in addition to these surfaces, the above ARL and TCO layer of the top sub cell are also patterned. All proposed cases have been demonstrated in Fig. 4a. In following simulations, the grating patterns have been considered as function $g(y)=L_{g}cos[\frac{\pi y}{\beta W}]$ for $y< |\frac{\beta W}{2} |$. Where, $W=350\;{\text{nm}}$, $\beta$ and $L_{g}$ are introduced as structural characteristics of grating shape (Fig. 4b). $(\beta W,L_{g})$ nm has been assumed (300, 90), (280, 85), (250, 80) and (220, 75) nm for the top of ARL and TCO layer of two sub cells, above *ETM* of the bottom sub cell, both sides of $MASnI_{3}$ layer and back-contact/HTM interface, respectively. The fabrication conditions were taken into account when choosing these grating shape structural characteristics. In these studies, the thicknesses of the ARLs and active layers have remained constant in comparison to the case (IV). The optical properties of the proposed cases (V)–(VIII) are compared to case (IV) in this section as shown in Fig. 10a,b.

The major changes are related to longer wavelengths of sunlight by creating nano-photonic structures above the top two layers of the bottom sub cell (case (V)). This is because shorter wavelengths are absorbed mainly in the top sub cell before reaching these structures. The absorption properties of the WBG perovskite layer are nearly unchanged when case (V) is compared to case (IV). Also, there is no noticeable change in the absorption of the NBG perovskite layer at wavelengths less than 660 nm. Only the absorption of the NBG perovskite layer is improved at wavelengths ranging about 670–710 and 810–960 nm. Since the incident light is entered to the bottom sub cell in non-vertical direction, the optical path-length is increased inside the $MASnI_{3}$ layer. Therefore, the absorption enhancement is observed in the NBG perovskite layer in these ranges. Because the light is trapped in the device, the total reflection losses are decreased at these wavelengths (comparison of blue and red lines in Fig. 10a,b).

Using nano-photonic structures above the top two layers of the top sub cell (case (VI)), not only longer wavelengths but also shorter wavelengths are affected. By comparing case (VI) with case (IV), the total reflection losses are reduced at the wavelength rage of 370–440 nm. This means that a smaller percentage of light is left the structure. Hence, the incident light is captured inside the device. In fact, these wavelengths of light are absorbed in the WBG perovskite layer by the collision with these structures, which increases the path-length of the light entering the structure. Significant changes in the first half of the absorption spectrum are not created in the NBG perovskite layer because the 370–440 nm wavelengths affected by these structures are absorbed by the WBG perovskite layer and not entered into the NBG perovskite layer. However, longer wavelengths are affected in the absorption and reflection spectra. By increasing the optical path-length, absorption enhancement can be observed at wavelength range from 870 to 930 nm. Also, the reduction of reflection losses happens due to light-trapping in this range. However, the reflection losses are slightly increased in the second half of the spectrum, except in the range of 870–930 nm, which is referred to as the DI between incident light and reflected light from the surfaces. Consequently, the absorption is decreased in the NBG perovskite layer (comparison of blue and green lines in Fig. 10a,b).

As shown in Fig. 10b, the most reflection losses are related to the second half of the spectrum. Since this range corresponds to the sensitivity range of absorption in the NBG perovskite layer, a part of the entered sunlight into this layer has not been absorbed and it has left the device. In order to increase the absorption ability, we exploited nano-plasmonic structures. To use the back-scattering effects of the nano-plasmonic structure, the size of the pattern located at the HTM/back-contact interface is assumed to be greater than 50 nm. Because the *HTM* layer is 50 nm thick, all top layers of the Ag-back contact in the bottom sub cell are also patterned without changing the thickness of the ARL and absorber layer. Thus, both nano-photonic and nano-plasmonic structures are employed. According to the absorption and the reflection spectra of cases (V) and (VII) (red and orange lines in Fig. 10a,b), no noticeable change is observed in the absorption of the WBG absorber layer, the first half of the absorption spectrum of the NBG absorber layer, and the first half of the total reflection. However, in the second half of the absorption spectrum related to the absorber layer of the bottom sub cell, the absorption enhancement occurred at wavelengths ranging of 630–710 and 740–970 nm. It is due to the scattering mechanisms caused by nano-photonic and nano-plasmonic structures. Nano-texture structures increase the optical path-length. Therefore, absorption of the NBG perovskite layer is enhanced. Also, total reflection losses are reduced in these ranges, implying that sunlight rarely leaves the device; it remains in the device due to the forward-scattering mechanism in the *MASnI*3 layer’s top surfaces, the back-scattering mechanism at the HTM/back-contact interface, and the changing angle of incoming light to total reflection angle at the *MASnI*3 layer/HTM interface.

By combining two cases (VI) and (VII), the features of these two cases are incorporated. This structure is introduced as case (VIII). As expected, similar behavior can be seen in the absorption spectrum of the WBG perovskite layer in cases (VI) and (VIII) (green and violet lines in Fig. 10a). Comparing the absorption spectrum of case (VII) with case (VIII) (orange and violet lines in Fig. 10a) shows that the absorption is improved at wavelengths ranging about 370–440 nm (the first range) and 870–930 nm (the second range). These changes are related to the absorption spectrum of WBG and NBG perovskite layers, respectively. The absorption enhancement in the first range is because the incident light collides with nano-texture structures located above the top sub cell and its direction is changed. Since it is moved non-perpendicularly, the length of the optical path is increased and the incident light is trapped inside the device. Thus, these changes have affected on the absorption spectrum of the WBG perovskite layer. However, embedded nano-photonic structures above the top sub cell have no effect on first half of the absorption spectrum of NBG perovskite layer. Because the first range is in the range of absorption sensitivity of WBG perovskite layer and it is absorbed here. Therefore, a greater percentage of this range is not reached to the NBG perovskite layer. As expected, because the sunlight is not captured in the WBG perovskite layer at the second range, therefore, entered into the bottom sub cell and improved the absorption of NBG perovskite layer. As a result, the total reflection losses at the first and second ranges are reduced because part of the incident light does not leave the device (comparison of orange and violet lines in Fig. 10b). In contrast, the total reflection loss has been increased in the parts of spectrum (at wavelength ranges of 750–780, 820–870 and 940–960 nm), as mentioned, due to the DI of incident and reflected lights in the top surfaces of device. Therefore NBG perovskite layer absorption decreased at these wavelengths, but the WBG perovskite layer absorption was unaffected by this increase in reflection loss.

### Electrical properties of all proposed cases

**Figure 11:**
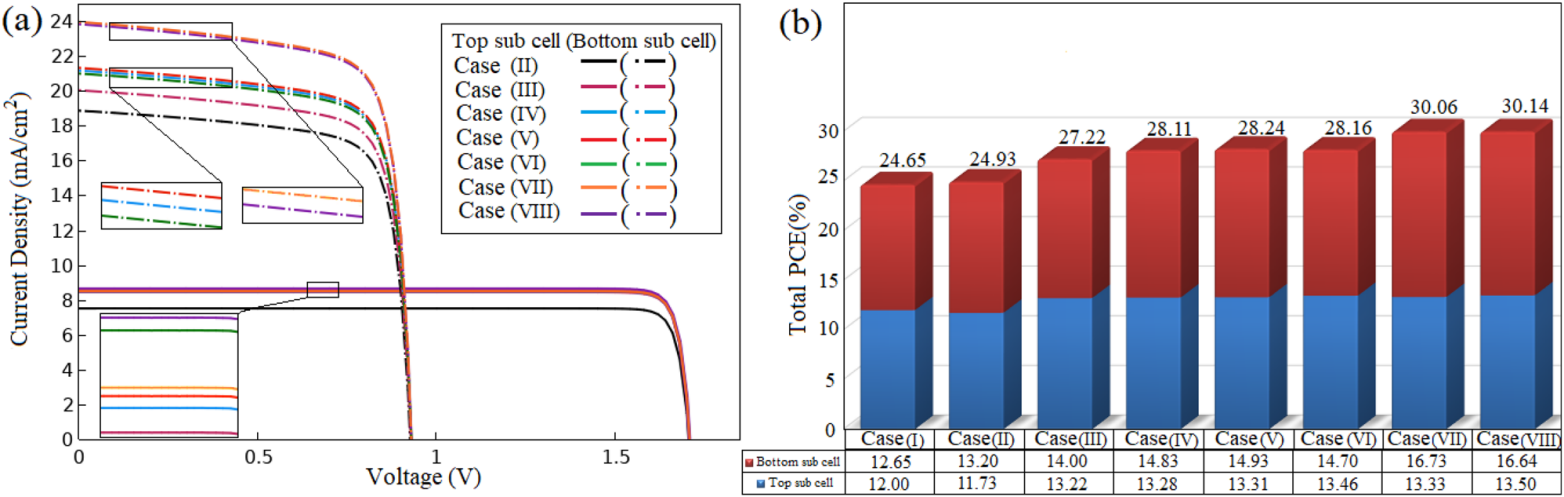
(**a**) The current-voltage characteristics of the proposed cases (II)–(VIII). (**b**) The *PCE* of each sub cell and the 4T structure for all proposed cases.

In this section, the electrical properties of cases (II)–(VIII) are studied and compared with the reference structure. Since the sub cells are electrically separated in the 4T configuration, the electrical properties of two sub cells are separately investigated. Figure 11a illustrates the electrical simulation results of the J–V characteristic for the top and bottom sub cells of cases (II)–(VIII). All electrical parameters of these proposed structures and reference structure including $J_{sc}$, $V_{oc}$, *FF* and *PCE* have been extracted from J–V curves (Figs. 3b and 11a) given in Table 1 for the top and bottom sub cells. To calculate the *PCE* of the 4T device, the *PCE* of the top and bottom sub cells is summed.

**Table 1:** The output electrical parameters of the top and bottom sub cells for all proposed APTSCs.

Parameter (top/bottom sub cell)	$J_{sc}\;({\text {mA}}/{\text {cm}}^{2})$	$V_{oc}\;({\text {V}})$	*FF*	$PCE (\%)$
case (I)	7.68/18.12	1.70/0.93	0.92/0.75	12.00/12.65
Case (II)	7.51/18.92			11.73/13.20
Case (III)	8.44/20.03			13.22/14.00
Case (IV)	8.48/21.16			13.28/14.83
Case (V)	8.50/21.31			13.31/14.93
Case (VI)	8.60/20.99			13.46/14.70
Case (VII)	8.51/23.95			13.33/16.73
Case (VIII)	8.63/23.83			13.50/16.64

According to Table 1, $V_{oc}$ and *FF* values are approximately constant in the top and bottom sub cells of all cases because these parameters do not depend on the concept of absorption enhancement, which is considered in this work. Because of the lower absorption density and photo generation rate in the WBG perovskite layer, the $J_{sc}$ and *PCE* parameters in case (II) are decreased to $7.51\;{\text {mA}}/{\text {cm}}^{2}$ and $11.73 \%$, respectively, when compared to the reference case. However, $J_{sc}$ and *PCE* values are boosted in the bottom sub cell. Due to the elimination of the parasitic absorption related to the buffer layer, absorption enhancement has occurred in the NBG perovskite layer, where the generation of carriers is increased. Therefore, $J_{sc}$ is reached to $18.92\;{\text {mA}}/{\text {cm}}^{2}$. Consequently, *PCE* of the bottom sub cell is increased to $13.20 \%$. In case (III), $J_{sc}$ and *PCE* values of the top and bottom sub cells are obtained $8.44\;{\text {mA}}/{\text {cm}}^{2}$, $13.22 \%$, $20.03\;{\text {mA}}/{\text {cm}}^{2}$ and $14.00 \%$, respectively. By reducing reflection from above the top sub cell, absorption in both sub cells is improved, which means the creation of electron–hole pairs is increased. As a result, $J_{sc}$ and *PCE* values are increased to $12.38 \%$ and $12.70 \%$ in the top sub cell and $5.87 \%$ and $6.06 \%$ in the bottom sub cell, respectively, in comparison to case (II). As expected, noticeable effects are only observed in the electrical features of the bottom sub cell when cases (III) and (IV) are compared. Therefore, $J_{sc}$ is reached $21.16\;{\text {mA}}/{\text {cm}}^{2}$ and *PCE* is increased to $14.83 \%$. This is because the $G_{tot}$ value is enhanced in the bottom sub cell. $J_{sc}=8.50\;{\text {mA}}/{\text {cm}}^{2}$ and $PCE=13.31 \%$ for the top sub cell and $J_{sc}=21.31\;{\text {mA}}/{\text {cm}}^{2}$ and $PCE=14.93 \%$ for the bottom sub cell are achieved in case (V). Due to no significant change in absorption of the WBG perovskite layer, a noticeable change has not happened in the electrical properties of the top sub cell of case (V) in comparison with case (IV). However, due to the absorption enhancement in the NBG perovskite layer, $J_{sc}$ and *PCE* parameters are increased $0.15\;{\text {mA}}/{\text {cm}}^{2}$ and $0.1 \%$, respectively. In case (VI), changes are observed in the electrical parameters of the both sub cells relative to case (IV). Because the absorption enhancement is occurred in $MAGeI_{3}$ layer, $J_{sc}$ and *PCE* values are increased $1.42 \%$ and $1.36 \%$ in the top sub cell, respectively. $J_{sc}$ and *PCE* parameters of the bottom sub cell are calculated $20.99\;{\text {mA}}/{\text {cm}}^{2}$ and $14.70 \%$, respectively. The reduction of these parameters compared with values in case (IV) is related to increasing reflection losses from above the top sub cell. By comparison the electrical properties of cases (V) with case (VII), as expected, $J_{sc}$ and *PCE* parameters are remained almost intact in the top sub cell. In the bottom sub cell, $J_{sc}=23.95\;{\text {mA}}/{\text {cm}}^{2}$ and $PCE=16.73 \%$ are obtained. The improvement in the values of these parameters is due to absorption enhancement in the NBG perovskite layer. In case (VIII), $J_{sc}=8.63\;{\text {mA}}/{\text {cm}}^{2}$ and $PCE=13.50 \%$ in the top sub cell and $J_{sc}=23.83\;{\text {mA}}/{\text {cm}}^{2}$ and $PCE=16.64 \%$ in the bottom sub cell are achieved. Due to the absorption enhancement of the WBG perovskite layer relative to case (VII), $J_{sc}$ and *PCE* of the top sub cell are increased. In the bottom sub cell, these parameters are reduced because the reflection losses happen in above the top sub cell at several wavelengths.

The *PCE* parameter of the top and bottom sub cells and 4T structure for all proposed cases is presented in Fig. 11b. The efficiency of the 4T structure is introduced as total *PCE*. The total *PCE* value of case (II) relative to reference case is increased $0.28 \%$ due to efficiency enhancement of the bottom sub cell. In case (III), this parameter is reached to $27.22 \%$. It is because the *PCE* value of the top and bottom sub cells is boosted $1.17 \%$ and $0.8 \%$, respectively. By comparing case (IV) with case (III), $0.89 \%$ improvement in the total *PCE* is observed. The main reason is the *PCE* enhancement of the bottom sub cell. The total *PCE* of cases (V) and (VI) is increased to $28.24 \%$ and $28.16 \%$ in comparison to case (IV), respectively. It is due to the increase in the summation of *PCE* value of the top and bottom sub cells. In case (VII), the increased efficiency results in a $1.82 \%$ total *PCE* boost, mainly because of efficiency enhancement of the bottom sub cell. By increasing the sum of the *PCE* values of two sub cells, the total *PCE* is $30.14 \%$ in case (VIII), which is about $0.1 \%$ higher than the case (VII). Finally, case (VIII) has the highest increase in total *PCE* when compared to the reference structure and is chosen as the best proposed case.

## Discussion

In this paper, a 3D coupled optical-electrical modeling was used to simulate single junction $MAGeI_{3}$- and $MASnI_{3}$-based PSCs with plastic substrates. Then, as a reference structure, a mechanically stacked 4T device of flexible APTSC was introduced and studied. The optical and electrical properties of two single junction PSCs and reference structure have been investigated and compared with each other in “Results”. The total *PCE* value of reference structure has been calculated $24.65 \%$. Cases (II)–(VIII) were then proposed to improve this structure’s *PCE*. First, The optical properties of all proposed cases have been studied and analyzed in the “The removal of the buffer layer”, “Addition of antireflection layer”, and “Periodic nano-texture structures to improve light absorption”. Finally, the electrical properties of the top and bottom sub cells, as well as their tandem structure, have been discussed in the “Electrical properties of all proposed cases”. By presenting case (II), the total *PCE* was increased by $1.14 \%$ when compared to the reference structure using the approach of reducing parasitic absorption by deleting the buffer layer. Cases (III) and (IV) have been proposed based on the use of *ARLs* above the top layer of two sub cells. The thicknesses of $ARL_{1}$ and $ARL_{2}$ were optimized to be 100 and 60 nm, respectively. The results show that the total reflection loss was lowered by closing the refractive indices of the top layers of each sub cell with the external ambience of the PSC (air layer) using optimized thickness *ARLs*. As a result, when comparing cases (II) and (IV), the total *PCE* value has increased by $12.76 \%$ due to absorption enhancement. In cases (V)–(VIII), the strategy of using nano-texture structures was used to improve absorption property and total *PCE* parameter. The optical and electrical properties of these proposed cases have been investigated and compared to case (IV). According to the simulation results, case (VIII) is the best structure based on the total *PCE* improvement due to absorption enhancement, which reached $30.14 \%$.

## Methods

The proposed 4T APTSC consists of two sub cells where both absorber layers are sandwiched between the *ETM* and the *HTM*. In the top and bottom sub cells, the *TCO* layer is used as the front contact. A metal oxide as a buffer layer and a *TCO* as a transparent back-contact are chosen for the semi-transparent top sub cell. Ag is chosen as the back reflector contact of the bottom sub cell. The materials are assumed to be isotropic.

The proposed TSC is studied using 3D FEM in two steps: electrical and optical parts to obtain the basic characteristics of each sub cell and the performance of the tandem device. FEM is a computational method for estimating partial differential equation boundary value problems. It’s a type of mathematical analysis that’s extensively utilized to solve a wide range of engineering problems. Using the mesh generation process, the entire domain of the structure is subdivided into small pieces called “elements”. The equations are solved numerically for each element. A user-controlled mesh is supplied for the proposed cases. The maximum element size is 65 nm, while the minimum element size is 2 nm. The maximum element growth rate with a curvature factor of 0.3 and the resolution of narrow regions are set to 1.35 and 0.85, respectively. All domains have a swept type mesh using the quadrilateral face meshing method. As a result, our simulation included 5904 elements.

### Optical simulation process

In this model, we used the Helmholtz equation to compute the electric field distribution in each layer, which is expressed as follows:

1 $$\begin{aligned} \nabla \times (\nabla \times {\mathbf{E }})-k_{0}^{2}(n(\lambda )-i k(\lambda ))^{2}{\mathbf{E }}=0 \end{aligned}$$

where *E* , $k_{0}$, $n(\lambda )$ and $k(\lambda )$ are the electric field distribution, the vacuum wave-vector of the incident light, the refractive index and the extinction coefficient as a function of the wavelength ($\lambda$), respectively. *n* and *k* in terms of $\lambda$ for all layers were taken from previous literatures^25,29,30,34–40,49,50^. We employed this equation for the wavelength range of 300–1050 nm. The incident light power of the optical wave ($P_{in}$) was applied to the AM1.5 G standard of the spectral irradiance, perpendicularly to the cross section of the device. For boundary conditions, ‘perfect electric conductor’ (PEC) was implemented for the back reflector (Ag) of the bottom sub cell to apply the reflected photons from its surface, and the Floquet ‘periodic boundary condition’ (PBC) was considered for all layer sides along the x and y directions except the Ag back-contact.

After computing a map of *E* in the whole domain of each sub cell by using Eq. (1), the absorbed power density for each wavelength could be obtained from the divergence of the Poynting vector as:

2 $$\begin{aligned} U_{ABS}(x,y,z,\lambda )=\frac{1}{2}\omega \varepsilon ^{''}E^{2}(x,y,z,\lambda ) \end{aligned}$$

where $\varepsilon ^{''}$ is the imaginary part of the dielectric permittivity and $\omega$ is the angular frequency of the light impinging on the device. By normalization of $U_{ABS}$ by $P_{in}$, the absorption density ($p_{ABS}$) can be obtained. The light absorption of each absorber layer as a function of the $\lambda$ is defined as:

3 $$\begin{aligned} A(\lambda )=\int p_{ABS}(x,y,z,\lambda )\,dV \end{aligned}$$

Using the S parameters, total reflection as a function of the $\lambda$ is calculated^33^.

By normalization of $U_{ABS}$ by the energy per photon ($\hbar \omega$) where $\hbar =\frac{h}{2\pi }$ (*h* is the plank’s constant), $U_{ABS}$ can be converted to the optical generation rate:

4 $$\begin{aligned} g_{opt}(x,y,z,\lambda )=\frac{\pi }{h}\varepsilon ^{''}E^{2}(x,y,z,\lambda ) \end{aligned}$$

Assuming that each absorbed photon generates one electron–hole pair, the total photogeneration rate will be given as follows:

5 $$\begin{aligned} G_{tot}(x,y,z)=\int _{300}^{1050}g_{opt}(x,y,z,\lambda )\,d\lambda \end{aligned}$$

### Electrical simulation process

This part is substantially based on the Poisson, the continuity and the drift-diffusion equations for evaluating current density–voltage characteristics. These equations are defined as:

6 $$\begin{aligned}&\nabla \cdot (\varepsilon _{0} \cdot \varepsilon _{r}\nabla \phi )=-\rho ;\,\rho =q(n-p+N_{A}-N_{D}) \end{aligned}$$

7 $$\begin{aligned}&\frac{\partial i}{\partial t}=\frac{1}{q}\nabla J_{i}+G_{i}-U_{i};\,i=n,p \end{aligned}$$

8 $$\begin{aligned}&J_{i}=-q\mu _{i}i\nabla \phi \pm qD_{i}\nabla i;\,i=n,p \end{aligned}$$

where $\phi$, q, $\varepsilon _{0}$ and $\varepsilon _{r}$ are the electrostatic potential, the electron charge, the vacuum permittivity and the relative permittivity, respectively. *n* and *p* are electron and hole concentrations, respectively, $N_{A}$ and $N_{D}$ are acceptor and donor densities, respectively, $\mu _{i}$ and $J_{i}$ are carrier mobilities and carrier current densities, respectively.

The functions $G_{i}$ and $U_{i}$ are the photogeneration and the recombination rates of electron and hole per unit volume, respectively. We considered $G_{n} = G_{p} = G_{tot}$. Where it is extracted from the optical part. Surface recombination, which is caused by defects in the layer interfaces, is not taken into account. Recombination mechanism in each layer, however, is considered. Shockley–Read–Hall (SRH) recombination is the dominant recombination mechanism in PSCs, according to previous studies^51,52^. Therefore, the model includes SRH recombination, which is defined as^53^:

9 $$\begin{aligned} R_{SRH}=\frac{np}{\tau _{n}(p+p_{1})+\tau {p}(n+n_{1})} \end{aligned}$$

The electron and hole densities when the quasi-Fermi level matches the trapped energy are $n_{1}$ and $p_{1}$, respectively, and the electron and hole lifetimes are $\tau _{n}$ and $\tau _{p}$, respectively. For the front contact of two sub cells and for the back reflector layer, ideal ohmic and Schottky contact conditions are applied with the surface recombination velocities ($S_{i}, i=n,p$) of carriers $10^{7}\;{\text {cm}}/{\text {s}}$.

All the numerical parameters used in the electrical model are indicated in Table 2^33,54–56^. Here, $\chi$ and $E_{g}$ are electron affinity and band gap, and $N_{C}$ and $N_{V}$ are effective density of states of conduction and valence bands, respectively.

**Table 2:** The electrical parameters of the modeled APTSC.

Parameter	$MAGeI_{3}$	$MASnI_{3}$	$TiO_{2}$	*Spiro*–*OMeTAD*	$MoO_{x}$
$\varepsilon _{r}$	10	8.2	9	3	12.5
$N_{C}\;({\text {cm}}^{3})$	$1 \times 10^{16}$	$1 \times 10^{18}$	$1 \times 10^{19}$	$1 \times 10^{20}$	$2.2 \times 10^{18}$
$N_{V}\;({\text {cm}}^{3})$	$1 \times 10^{15}$	$1 \times 10^{18}$	$1 \times 10^{19}$	$1 \times 10^{20}$	$1.8 \times 10^{19}$
$\mu _{n}/\mu _{p}$	$162 \times 10^{3}/101 \times 10^{3}$	1.6/1.6	20/10	2/0.01	25/100
$\chi$	3.98	4.17	4	2.45	2.5
$E_{g}$	1.9	1.3	3.2	3	3
$N_{A}/N_{D}\;({\text {cm}}^{3})$	$1 \times 10^{9}/1 \times 10^{9}$	$3 \times 10^{16}/-$	$-/5 \times 10^{18}$	$5 \times 10^{18}/-$	$1 \times 10^{18}/-$
$\tau _{n}/\tau _{p}$	25/25	25/25	5/2	0.1/0.1	5/5

The PCE of the solar cell is calculated as follows:

10 $$\begin{aligned} PCE=\frac{FF\,J_{sc}\, V_{oc}}{P_{in}} \end{aligned}$$

where $J_{sc}$, $V_{oc}$ and *FF* are the short-circuit current density, the open-circuit voltage and fill factor, respectively. The fill factor can be expressed as:

11 $$\begin{aligned} FF=\frac{V_{mp}\,J_{mp}}{V_{oc}\,J_{sc}} \end{aligned}$$

where $V_{mp}$ and $J_{mp}$ are the voltage and current density at the maximal power point that are obtained from the J–V characteristic.
